# Prevalence of injury and associated factors among patients visiting the Emergency Departments of Amhara Regional State Referral Hospitals, Ethiopia: a cross-sectional study

**DOI:** 10.1186/s12873-015-0044-3

**Published:** 2015-08-25

**Authors:** Debrework Tesgera Bashah, Berihun Assefa Dachew, Bewket Tadesse Tiruneh

**Affiliations:** Department of Nursing, College of Medicine and Health Sciences, University of Gondar, Gondar, Northwest Ethiopia; Department of Epidemiology and Biostatistics, Institute of Public Health, College of Medicine and Health Sciences, University of Gondar, Gondar, Northwest Ethiopia

## Abstract

**Background:**

Injury significantly affecting the health and well-being of the society.

The prevalence tends to be higher in low income countries. The aim of this study was to assess the prevalence of injury and associated factors among patients visiting emergency departments of Amhara Regional State referral hospitals.

**Methods:**

Institution based cross sectional study was conducted from March to April 2014. The systematic random sampling technique was employed to select the study participants. The data were collected using an interviewer administered questionnaire. Bivariate and multivariate logistic regressions were performed to identify factors associated with injury. Odds ratios with 95 % confidence interval were computed to determine the level of significance.

**Result:**

The prevalence of injury was 55.6 %. Being male (AOR = 2.8; 95 % CI, 1.79-4.47), monthly income less than 34.2 USD (AOR = 1.89; 95 % CI, 1.03–3.46), being age between 20 to 44 years (AOR = 2.25; 95 % CI, 1.06–4.81), being a daily laborer (AOR = 6.27; 95 % CI, 2.38-16.47), being a farmer (AOR = 2.9; 95 % CI, 1.31-6.41) and being a substance user (AOR = 2. 16; 95 % CI, 1.18-3.96) were significantly associated with injury.

**Conclusion:**

The prevalence of injury was high. Being male, age 20 to 44 years, income < 34.2 USD, being a daily laborer, being a farmer and alcohol use were factors associated with injury. Hence, appropriate injury prevention strategy should be designed in order to lessen the magnitude of injury.

## Background

Injury has profound impact on the individual and the entire society. It is becoming one of the leading causes of premature death and disability. It contributes to 16 % of the global burden of disease and 10 % of mortality [1–3]. The burden of injury is highest between the ages of 15 and 45 years [4–6]. This age group is believed to be the most productive, and injury will in turn have an impact on growth and economic development of a nation.

According to World Health Organization (WHO), low and middle income countries (LMICs) share more than 90 % of injury cases. Of which Africa regions contribute for about 21 %, mainly the sub Saharan countries [3]. Reports from South Africa and Zimbabwe revealed that injury accounted for the largest proportion of all deaths and morbidities [7]. Findings from East Africa countries such as Kenya, Sudan and Tanzania demonstrate that there is a significant growing burden of traumatic injuries [2, 8].

In Ethiopia, like other developing countries, injury is a common public health problem [9]. Research conducted at university of Gondar referral hospital revealed that injury accounts for 25 % of surgical cases [10].

Although injuries are known to be preventable, still it continues to be the a widespread health problem [3, 4]. In Ethiopia, despite government efforts to reduce road traffic injuries, injury is increasing at an alarming rate and constitute around half of all surgical emergencies [11, 12]. On the other hand, lack of sufficient data about its magnitude leads to underestimation of injury burden [13]. Thus to design effective prevention strategies, there is need of findings about the magnitude of injury and its associated factors. Therefore, the aim of this study was to determine the magnitude of injury and its associated factors among patients visiting the emergency departments of Amhara regional 74 state referral hospitals, Ethiopia.

## Methods

An institution based cross-sectional study was conducted from March to April 2014 in Amhara national regional state referral hospitals. Amhara national regional state is one of the federal republic of Ethiopia regional states, with an estimated area of 159,173.66 square kilometers. Based on the Central Statistical Agency (CSA) of Ethiopia 2007, the region has an estimated total population of 20,136,000, consisting of 10,060,000 men and 10,076,000 women. Eighty eight percent of the population is estimated to be rural inhabitant, while 12 % are urban dwellers. Health and health related indicators published by the federal ministry of health (FMoH) reveals that, there are 20 hospitals, including the five referral hospitals in the region [14].

In this study all patients who visited the emergency departments of the three referral hospitals of Amhara regional state (both injured and non-injured) were included in the study. Whereas those patients who were seriously ill and in need of urgent transfers to another health institution were excluded. Injury cases were operationally defined as patients who encountered sustained physical trauma prior to appearing to the emergency department of the referral hospitals.

The sample was calculated using single population proportion formula to obtain the sample size needed to estimate the prevalence of injury in the population visiting referral hospitals of Amhara regional state. It was determined by the assumptions of 95 % confidence level, 4 % margin of error, and taking the prevalence of injury 8.2 % [15]. Taking into account design effect of two and considering 15 % non response rate, the final simple size became 416. Among five referral hospitals in Amhara region (Dessie, Debre-99 Birhan, Debre-Markos, Felege-Hiwot and university of Gondar hospital) three referral hospitals namely Debre-Birhan hospital, Felege-Hiwot hospital and University of Gondar hospital were taken by a lottery method. The participants were included proportionally by considering the daily patient flow of emergency departments of the selected referral hospitals; that is participants from University of Gondar Hospital, 137 from Felege-Hiwot hospital and 122 participants from Debre-Birhan hospital. Then systematic sampling technique was used to select study participants and every third patient was selected until the desired sample size was obtained.

Semi-structured questionnaire adapted from WHO injury surveillance document was used to collect the data. The tool was validated in low and middle income countries. It was modified to fit the study population. Pretest was conducted to check compatibility of the tool. The questionnaire was first prepared in English and then translated into the local language (Amharic), and back to English to ensure consistency. This questionnaire gathered data on injury events, nature, severity and patient disposition. Field supervisors reviewed the questionnaires daily.

Data cleaning was carried out by the principal investigator. Data were checked for completeness, coded and entered to Epi-info version 3.5.1 statistical software and was exported to SPSS (Statistical Package for Social science) windows version 20 for further analysis. Frequencies and cross tabulations were used to summarize descriptive statistics of the data. Bivariate logistic analysis was used primarily to check variables which have associations with the dependent variable. Variables found to have associated with the dependent variable at p-value <0.02 were then entered into multiple logistic regression for controlling the possible effect of confounders and finally the variables with significant association at P-value < 0.05 were identified 124 on the basis of Odds Ratio with 95 % confidence interval.

Ethical clearance was obtained from Ethical review committee of University of Gondar (Reference Number 1038/06) and permission was secured from the head offices of the respective referral hospitals with a formal letter of cooperation. Each respondent was informed about the purpose of the study and informed verbal consent was obtained from each study participants before the actual data collection.

## Results

### Socio demographic characteristics of study participants

Among the 416 study participants, 414 responded to the interview making the response rate of 99 %. In this study, males outnumbered females by a sex ratio of 2: 1, (males 278 (67.1 %) and females 136 (32.9 %). The mean and SD of age of patients was 30 (±16) years. Majority 356 (86 %) of the study participants were Orthodox Christian. Regarding the marital status, 219 (53 %) of the participants were single. Among the participants 229 (55.3 %) were urban dwellers. The data about the educational level of the study subjects’ showed that 198 (47.8 %) have no formal education and 109 (26.3 %) have attended primary education. Among the 414 patients consented to interview, 98 (23.7 %) have monthly income less than 34.2 USD, and a similar proportion of the group had income ranging between 34.3 -73.4 USD (Table 1).

**Table 1:** Socio-demographic characteristics of the study participants visiting emergency departments (EDs) of Amhara regional state referral hospitals, 2014; (n = 414)

Variables	Frequency, n = 414	Percent
Sex		
Male	278	67.1
Female	136	32.9
Age in years		
<4	19	4.6
5-14	45	10.9
15-19	38	9.2
20-24	66	15.9
25-44	165	39.9
45-64	63	15.2
≥ 65	18	4.3
Religion		
Orthodox Christian	356	86.0
Muslim	33	8.0
Protestant	18	4.3
Other	7	1.7
Marital status		
Married	167	40.3
Single	219	52.9
Others	28	6.8
Educational level		
No education	198	47.8
Primary education	109	26.3
Secondary and above	107	25.8
Monthly income in dollar		
≤ 34.2	98	23.7
34.3 - 73.4	98	23.7
>73.4	218	52.6
Occupation		
Civil servant	49	11.8
Constr. Worker	6	1.4
Driver/assistant	16	3.9
Daily laborers	47	11.4
Farmer	123	29.7
House wives	25	6.0
Student	82	19.8
Trader of any kind	57	13.8
Unemployed	9	2.2
Residence		
Urban	229	55.3
Rural	185	44.7

### Prevalence of injury

**Fig. 1:**
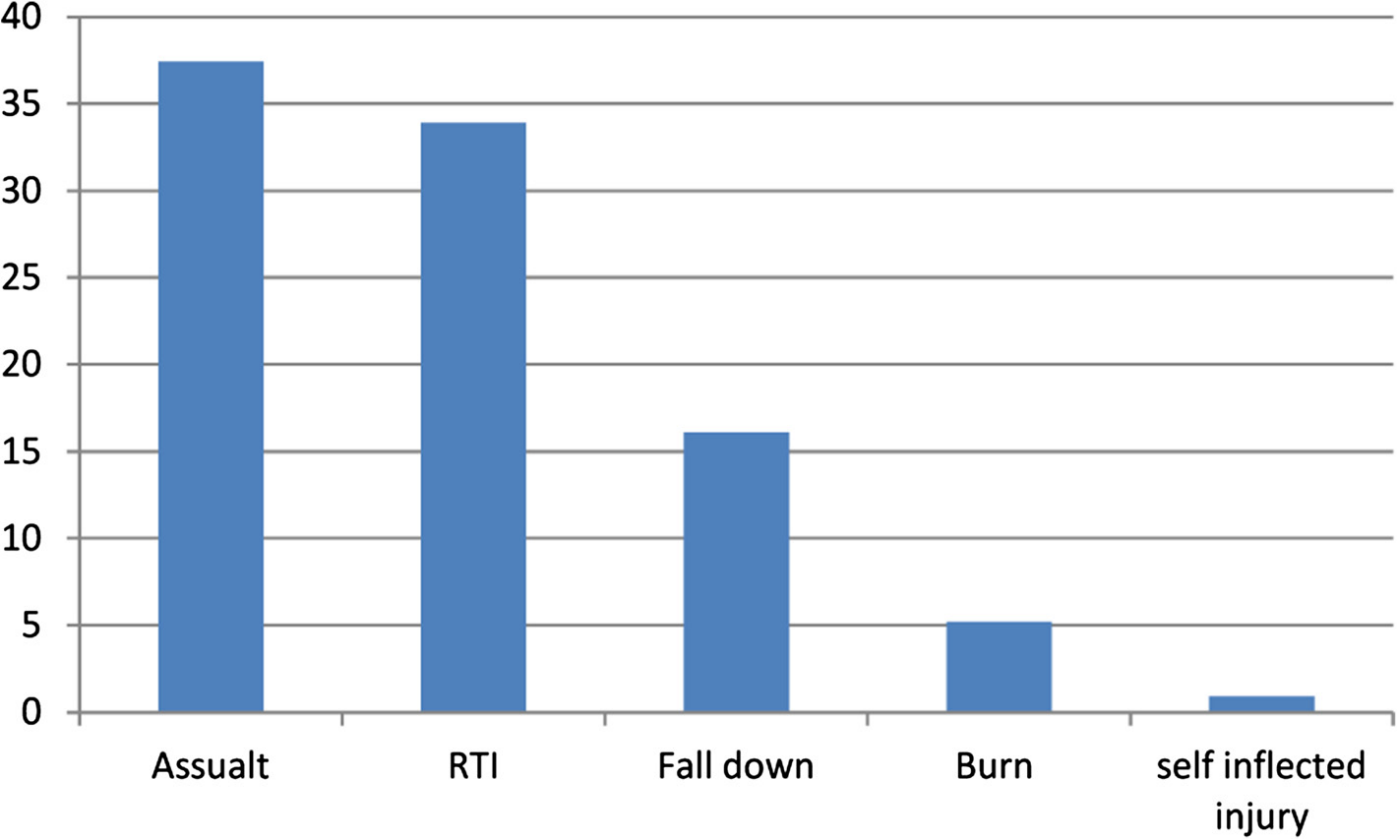
Mechanism of injury in patients visiting the emergency department of Amhara regional state referral hospitals, 2014 (n = 230)

The prevalence of injury in emergency departments of the referral hospitals, was found to be 55.6 % with 95%CI (50.7-60.4 %). Of which 116 (50.4 %) cases were from the University of Gondar hospital, 59 (25.7 %) cases from Felege Hiwot hospital and the rest 55 (23.9 %) of cases were from Debre Birhan hospital. Unintentional injuries were the primary cause for 165 (71.7 %) of cases. Forty six percent of the unintentional injury was contributed by Road Traffic Injury (RTI). Regarding the mechanism of injury, assault was cause for 86 (37.4 %) and RTI for 78 (33.9 %) of patients (Fig. 1).

Concerning the outcome of injury and the type of care given, 99 (43 %) of patients suffered moderate injury that needs skilled treatment such as suturing of wounds and stabilization of fractures. The other 78 (33.9 %) had a severe injury requiring highly specialized skills of medical and surgical management and the rest 53 (23 %) encountered minor superficial injury. Among the injured patients 161 (70 %) were admitted to hospital 66 (28.7 %) patients were treated and discharged.

**Fig. 2:**
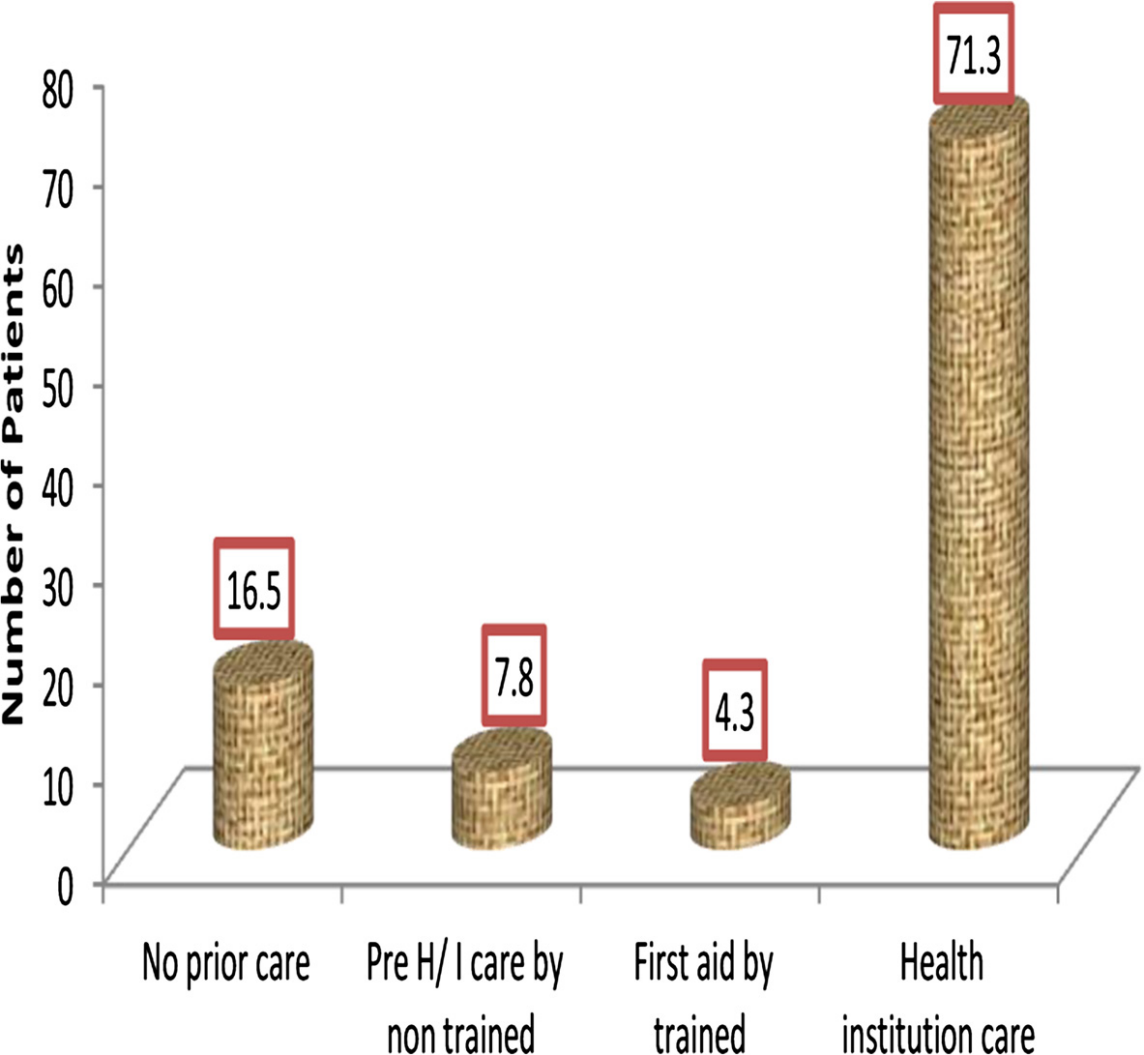
Type of care given prior to arrival to referral hospital among patients visiting the emergency department of Amhara region referral hospitals, 2014, (n = 230)

The injury resulted fracture in 95 (41.3 %) of patients, 68 (29.6 %) had cuts. One hundred sixty four (71.3 %) had got care at different level health institutions prior to their presentation to the emergency department of referral hospitals (Fig. 2).

The higher proportion 106 (46.1 %) of injury occurred on the street and the least proportion 15 (6.5 %) occurred at school and sports area. The mode of transport used by the casualties was varied among the patients; forty five (19.6 %) were coming by walking or carried by people, 132 (57.4 %) used a taxi or other private car, while 9 (3.9 %) came by police transport 168 and only 44 (19 %) got an ambulance service to reach the referral hospitals.

### Factors associated with injury

In the bivariate analysis the age of respondents, sex, occupation, residence, substance use, educational level and income were identified to be significantly associated with injury. However, only age 20-44 (AOR = 2. 25; 95 % CI, 1.06–4.81), sex: male (AOR =2. 83; 95 % CI, 1.79-4.47), income less than 34.2 USD (AOR = 1.89; 95 % CI, 1.03–3.45), occupation; daily laborers (AOR = 6.27;95 % CI, 2.38-16.47) and farmers (AOR = 2. 90; 95 % CI, 1.31-6.41) respectively and substance use (AOR = 2. 16; 95 % CI, 1.18-3.96) were remained significantly associated with injury at the multiple logistic regression analysis (Table 2).

**Table 2:** Bivariate and multivariate out put on factors affecting prevalence of injury in Amhara regional state referral hospitals, 2014

Variables	Non injured	Injured	Bi vairate analysis COR(CI)	Multivariate analysis AOR (CI)
Sex				
Male	101	177	2.7(1.8-4.18)*	2.8(1.79-4.47)*
Female	83	53	1	1
Age				
≤ 19	35	67	1.8(1.12-3.130)	1.3(.72-2.10)
20 – 44	101	130	2.7(1.52-5.08)*	2.2(1.06-4.81)*
> 45	48	33	1	1
Marital status				
Married	81	86	1	
Single	88	131	1.4( .93-2.10)	
Others	15	13	.81(.36-1.82)	
Income ($)				
≤ 34.2	85	141	1.84(1.24-2.73)*	1.89(1.03-3.45)*
> 34.2	99	89	1	1
Educ. level				
Illiterate	87	111	1.04(.70-1.53)	
Litrate	97	119	1	
Alcohol use				
Yes	21	49	2.1(1.20-3.65)*	2.16(1.18-3.96)*
No	163	181	1	1
Occupation				
Daily labourers	11	36	7.42(2.99-18.39)*	6.27(2.38-16.47)*
Student	33	49	3.36 (1.58-7.13)	2.19(.84--5.68)
Farmers	49	74	3.42(1.68-6.94)*	2.9(1.31-6.41)*
Trader	33	24	1.64(.73-3.68)	1.5(.66-3.68)
Others	24	32	3.02(1.35-6.76	2.48(.91- 6.77)
Civil servants	34	15	1	1
Residence				
Rural	72	113	1.5(1.01-2.22)*	1.08(.53-1.10)
Urban	112	117	1	1
Psych active.				
Yes	11	27	2.09(1.00-4.34)*	2.14(.97-4.75)
No	173	203	1	1

*Significantly associated at p-value <0.05. ^∞^Variables included in the multivariate analysis were: age, sex, marital status, income, educational level, alcohol use, occupation, residence, psycho active substance use

## Discussion

The study found that 55.6 % (at 95 % CI 50.7-60.4 %) of emergency visits were due to injury. The result of this study is much higher than the community based study of Tanzania (4.3 %) [16]. This difference may be due to differences in the study setting, since current study conducted in the hospital setting, mainly the emergency departments.

The finding of this paper is approximately twice as reports of hospital based data of Cameroon 27 % [17]. A possible explanation of the difference may be the current study is in the emergency department while the Cameroon study was a review of ward logs for injury data completeness. The result of this study is slightly lower than studies conducted in Uganda 70.4 % [18]. This may be due to the fact that, the current study was done in regional referral hospitals, while the Uganda’s study was done in its capital city (Kampala). The discrepancy may also be explained by difference in student population and the data source, since the current paper used primary data. The finding of this work is lower than reports from Addis Ababa, Ethiopia, which is 70.5 % [9]. The difference may be due to the difference in the duration of the study.

However, the current finding is higher than findings of Jimma University specialized Hospital (JUSH) 8.2 % and Tikur Anbessa Specialized referral Hospital (TASRH) which is 32.5 % [15, 19]. The Possible explanation may be the difference in the study setting. The current study is conducted only in the emergency departments while the JUSH and TASRH studies included all departments in the hospital.

In this study unintentional injuries contribute for more than two third 165 (71.7 %) of the injury cases. This figure is supported by the study conducted in Addis Ababa, which showed 76.4 % of cases were unintentional [20]. Road traffic injury (RTI) is one of the most common injuries contributed to 46 % of unintentional injuries. This may be due to lack of well established pedestrian’s road in the region. Furthermore, there is no separate road for domestic animals in the region and also most of the main roads are not supported by a traffic light.

The cause of injury was assaulted in 37.4 % cases followed by RTI (33.9 %). This finding is similar to findings in Jimma University Specialized hospital in which assault contributed to 30.9 % of injury cases followed by RTI 30.3 % [20]. However, it contradicts with findings of Tikur Anbessa in which RTI ranks first 38.3 %, followed by striking /hit by a person 31.5 %. This may be due to the fact that regional referral hospitals serve for both referral cases and non referral patients. So that, any injury patient of any cause can visit the emergency department. Another explanation may be regional states are less crowded with vehicles than the Addis Ababa city so that RTI can be high in Addis Ababa.

The primary places for the occurrence of injury were street for 46.1 % cases followed by home and home environment (27 %). This is supported by reports made from the state of Qatar, JUSH, and Tikur Anbessa Hospital [21, 22]. This study showed that 43 % of patients suffered moderate injury that needs skill treatment (eg. Stabilization of fractures, suturing of the wound) followed by severe injury which is 33.9 % and the rest 20.9 % had minor injuries. This finding is similar to findings of JUSH [22] which classified 41.3 % as moderate and 26 % as having a minor superficial injury. However study in Addis Ababa reported moderate injury as 71 % sever as 14.6 %.

This study found that, the sex was significantly associated with injury, i.e. Males were 2.8 times more likely to sustain injury. This is supported by the findings of other similar studies [4, 5, 20, 23]. This may be explained by the similarity in male travelling risk, emotional and risk taking behavior. Age was also seen to be associated with injury and the odds of injury were 2.3 times more likely in participants’ ages between 20-44 years. This is similar to findings of WHO, Quatar, Addis Ababa, JUSH and Tikur Anbessa [5, 20, 21]. Possible explanation may be that this age group is the active working years of life, time for practicing independent life out of parental supervision. In turn, this may predispose them to use substances. This magnifies the possible economic impact of injury as the productive age group of the society is primarily affected.

The study has also indicated that farmers were 2.9 times and daily laborers were 6 times more likely to be injured as compared to civil servants. The result is in line with study in Jimma that reveals more than a quarter (27.8 %) of injury patients were farmers [22]. This may be due to the fact that minimum 241 or no safety measures are used by farmers as well as the day laborers. Another explanation may be most of the civil servants live relatively stable and less risky life.

The study has also indicated that increased risk of injury with decreased income level. Patients who have a monthly income < 34.2 USD were 1.89 times more likely to visit the ED because of injury. This is supported with study in Minnesota that states monthly income < 416 USD increases the risk of injury by 2.85 times [24]. This may be because of their low income level; they may try to find other means of income which may have injury risk, or they may have impaired thought process, stressed and lack concentration in their activity [25].

Substance use was also shown to be another important variable significantly associated with injury. Those patients who had reported to use alcohol were 2.16 times more likely to present for injury. This is almost similar to findings of Minnesota that reported alcohol use increased injury risk by 1.79 times [24]. The observed result is supported by reports of WHO that showed, up to 45 % of injured patients reported consumption of alcohol prior to their injury [26]. Also reviews from Cape Town and Canadian study reported drunkenness (social risk taking) was positively associated with injuries [26, 27].

### Limitation of the study

Since the study was conducted in hospitals, merely in emergency departments of the referral hospitals it cannot be generalized to the population living in the catchment area and prevalence of injury may be overestimated. Furthermore the time of year may also create a selection bias in the results, as injury patterns have been shown to vary seasonally; this could affect other diseases that typically present to the emergency room, limiting the interpretation of the results. In addition, since the study addresses only the human factor of injury the other factors remained untouched.

## Conclusion

The study found that the magnitude of injury is considerably high in Amhara regional, state referral hospitals and also age between 20 to 44, being male, monthly income <34.2$, being a farmer, being a day laborer, and alcohol use was significantly associated with injury.

## Abbreviations

AOR: Adjusted odds ratio
C I: Confidence interval
CSA: Central statistical agency
D ALYs: Disability adjusted life years
E D: Emergency department
FMoH: Federal ministry of health
JUSH: Jimma university specialized hospital
LMIC: Low and middle income countries
MOH: Ministry of health
RTI: Road traffic injury
SPSS: Statistical package for social sciences
SSA: Sub Saharan Africa
USA: United States of America
WHO: World Health Organization
